# Distinct effects of β1 integrin on cell proliferation and cellular signaling in MDA-MB-231 breast cancer cells

**DOI:** 10.1038/srep18430

**Published:** 2016-01-05

**Authors:** Sicong Hou, Tomoya Isaji, Qinglei Hang, Sanghun Im, Tomohiko Fukuda, Jianguo Gu

**Affiliations:** 1 Division of Regulatory Glycobiology, Institute of Molecular Biomembrane and Glycobiology, Tohoku Pharmaceutical University, Sendai, Miyagi, 981-8558, Japan

## Abstract

An aberrant expression of integrin β1 has been implicated in breast cancer progression. Here, we compared the cell behaviors of wild-type (WT), β1 gene deleted (KO), and β1 gene restored (Res) MDA-MB-231 cells. Surprisingly, the expression of β1 exhibited opposite effects on cell proliferation. These effects were dependent on cell densities, and they showed an up-regulation of cell proliferation when cells were cultured under sparse conditions, and a down-regulation of cell growth under dense conditions. By comparison with WT cells, the phosphorylation levels of ERK in KO cells were consistently suppressed under sparse culture conditions, but consistently up-regulated under dense culture conditions. The phosphorylation levels of EGFR were increased in the KO cells. By contrast, the phosphorylation levels of AKT were decreased in the KO cells. The abilities for both colony and tumor formation were significantly suppressed in the KO cells, suggesting that β1 plays an important role in cell survival signaling for tumorigenesis. These aberrant phenotypes in the KO cells were rescued in the Res cells. Taken together, these results clearly showed the distinct roles of β1 in cancer cells: the inhibition of cell growth and the promotion of cell survival, which may shed light on cancer therapies.

Integrins comprise a group of transmembrane heterodimeric proteins consisting of α and β subunits^1^ that drive most of the interactions between cells and the extracellular matrix (ECM). β1 integrin, which constitutes the largest subgroup of integrins, is aberrantly expressed in human breast carcinoma and contributes to diverse malignant phenotypes, including epithelial-to-mesenchymal transition (EMT), metastasis, and angiogenesis^2^,^3^,^4^. In addition to the roles of β1 integrin in cancer progression, growing evidence has highlighted its relationship with tumor resistance to therapeutic modalities^5^,^6^. Due to its multiple important roles in breast cancer, the targeting of β1 is a promising strategy that can enhance therapeutic outcomes.

Several experimental models have shown that targeting β1 could partly attenuate aggressive tumor phenotypes in three-dimensional cell cultures and human breast cancer xenografts^7^,^8^,^9^. However, the effects of β1 on cell proliferation and cell survival in breast cancer cells are controversial, and the underlying mechanisms remain unclear. As a positive regulator, treatment with a functional blocking antibody against β1 is known to decrease cell proliferation and induce cell apoptosis^8^. In contrast, at least one study found that the functional blocking antibody had no inhibitory effects on cell growth, cell survival or capacity to form colonies in several breast tumor cell lines^10^. Therefore, a better understanding of the molecular mechanisms responsible for these differences is critical for the development of efficacious treatments for breast cancer.

The multiple downstream signaling pathways of β1, including FAK, PI3K and ERK/MAPK, coordinating signaling through receptor tyrosine kinases (RTKs), are involved in the modulation of tumor initiation, progression, and ultimately metastasis^2^,^11^,^12^,^13^. Although ample evidence has demonstrated that β1 plays critical roles in breast cancer, the targeting of β1 by using a monotherapy approach has not shown much benefit. Some possible mechanisms are involved in this phenomenon, such as the activation of intracellular protein kinase signaling pathways (e.g. PI3K and MAPK) and cross-talk between β1 and RTKs^14^,^15^. These mechanisms provide evidence that the biological events mediated by β1 are not limited to one signaling pathway, which highlights the fact that these signaling networks act dynamically and intersect with each other to control the physiological and pathological responses^14^. In addition, the dynamics of β1 signaling is further complicated by the cross-talk with RTKs, which is a crucial event in breast cancer progression^6^. Until just recently, the integrin-mediated dynamics of the regulation between different signal pathways have remained largely unknown.

Notably, the correct integration of signals from cell-ECM, cell-cell, and growth factor pathways is pivotal for a wide range of cellular biological functions, while deregulation of these signaling pathways results in a loss of tissue organization and contributes to tumorigenesis and progression^16^,^17^. β1 integrin integrates signals that maintain a balance of the biological functions in mammary tumor development primarily by appropriate interactions between cell-ECM and cross-talk with EGFR^6^. These signal integrations can also be achieved even when other signaling pathways are constitutively deregulated^15^,^18^. However, the roles of β1 in these processes remain unclear.

To solve these issues, here we investigated the biological functions of β1 in wild-type (WT) cells, the deletion of the β1 gene (KO), and the restoration of the β1 gene in KO (Res) MDA-MB-231 cells, and found that β1 exhibited opposite effects on cell proliferation that were dependent on cell densities: up-regulation of cell proliferation when cells were cultured under sparse conditions, and down-regulation of cell growth when cells were cultured under dense conditions. The abilities for cell survival were clearly decreased in KO cells, compared with those in WT and Res cells. Additionally, a treatment with AG1478, an inhibitor of EGFR, could more efficiently inhibit cell proliferation in KO cells than in WT cells. Thus, our study clearly showed the dynamic regulation by β1 for cell behavior, which may provide an underlying mechanism for the possibility of drug resistance due to β1 presence, and highlights the importance of combination treatment including β1 integrin and EGFR.

## Results

### Knockout of the β1 integrin gene altered cell morphology

**Figure 1:**
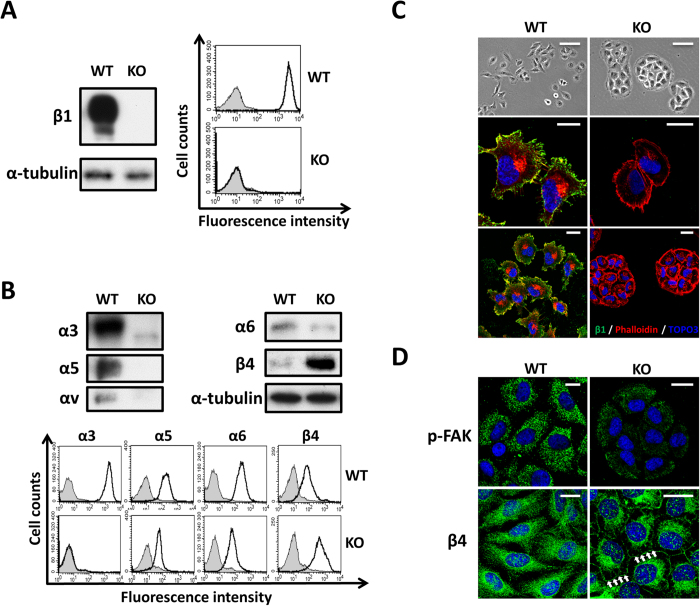
Knockout of β1 in MDA-MB-231 cells altered cell morphology and the expressions of other integrin subunits. (**A**) WT and KO cells were immunoblotted by anti-β1 antibody, α-tubulin was used as a loading control (left panel). WT and KO cells were collected and incubated with (bold line) or without (grey shadow) anti-β1, followed by incubation with Alexa Fluor 647 goat anti-mouse IgG subjected to flow cytometry analysis (right panel). (**B**) Cell lysates from WT and KO cells were immunoblotted with anti-α3, anti-α5, anti-α6, anti-αV and anti-β4 antibodies. α-Tubulin was used as a loading control (upper panel). Cells were collected and incubated with (bold line) or without (grey shadow) anti-α3, anti-α5, anti-α6 and anti-β4 antibodies, followed by incubation with Alexa Fluor 647 goat anti-mouse IgG subjected to flow cytometry analysis (lower panel). (**C**) Bright field pictures were taken to show the representative cell morphology. Scale bar, 50 μm (upper panel). WT and KO cells were stained with anti-β1 antibody, followed by incubation with fluorescent secondary antibody. Localization of F-actin was examined by staining with Alexa Fluor 546 phalloidin, the bar denotes 20 μm (middle and low panel). (**D**) The indicated cells were stained with anti-pFAK and anti-β4 antibody, followed by the incubation with fluorescent secondary antibody. Scale bar, 20 μm. The arrows indicate β4 integrin, the hemidesmosome maker, expressed in the cell-cell contact.

To explore the functional significance of β1 integrin, we established β1-KO MDA-MB-231 cells via a CRISPR/Cas9-based approach. Western blot and FACS analysis were performed to confirm the effective knockout of β1 in the cell line (Fig. 1A). As expected, the expression levels of β1-associated major α subunits were significantly decreased, but β4 integrin demonstrated a compensatory up-regulation in KO cells (Fig. 1B). During cell culture, we noticed that the KO cells lost the typical epithelioid morphology of WT cells, and showed a cell aggregation that appeared as a colony with a round shape (Fig. 1C upper panel). This phenomenon was also observed in the breast cancer cell lines treated with a blocking antibody against β1 antibody^15^. Consistently, immunofluorescent staining showed that KO cells exhibited cortical actin around the cell surface and decreased the formation of filopodia and lamellipodia (Fig. 1C middle and lower panel). Furthermore, the phosphorylation levels of focal adhesion kinase (FAK), a focal adhesion marker, were significantly decreased, while β4 integrin, a hemi-desmosome marker, showed an increase in the KO cells compared with the results seen in WT cells (Fig. 1D). These results indicated that β1-KO cells induced a disruption of focal adhesions and instead enhanced the cell-cell adherens junctions, which inhibited cell mobility.

### β1 integrin was essential for cell migration and the maintenance of mesenchymal phenotypes by stabilizing focal adhesions

**Figure 2:**
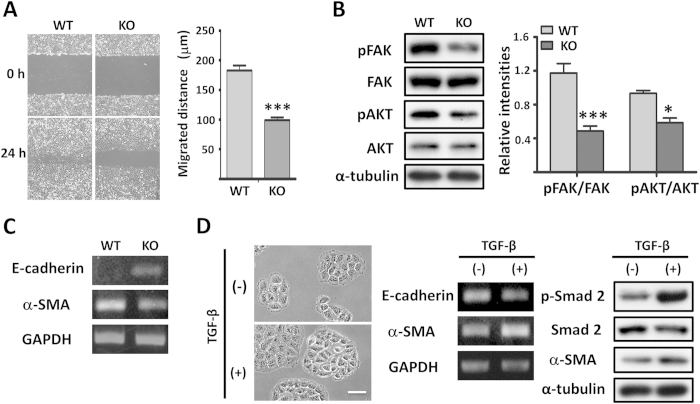
β1 was essential for malignant phenotype reversion and FAK/AKT signaling. (**A**) Cells were cultured until reaching more than 90% confluence. A scratch was made with a pipet in each well, and was photographed at 0 h and 24 h (left panel). Quantification of cell migrated distances are expressed as the means ± s.e.m from three independent experiments (***p < 0.001 by two-tail unpaired t-test) (right panel). (**B**) Cell lysates from WT and KO cells were immunoblotted by anti-pFAK, anti-FAK, anti-pAKT and anti-AKT antibodies. α-Tubulin was used as a loading control. The quantitative data are presented as the means ± s.e.m from three independent experiments (*p < 0.05, ***p < 0.001 by two-tail unpaired t-test). (**C**) RT-PCR analysis using total RNA extracted from WT and KO cells was carried out to examine the expression levels of E-cadherin and α-SMA. The expression level of GAPDH was used as a loading control. (**D**) Images of β1-KO cells that were untreated or stimulated with TGF-β for 48 h. Scale bar represents 50 μm (left panel). RT-PCR using total RNA extracted from indicated cells was carried out to detect the expression levels of E-cadherin and α-SMA. GAPDH was used as a loading control (middle panel). Representative western blots corresponding to the expression of pSmad2, Smad2 and α-SMA in TGF-β treated and untreated β1-KO cells, α-tubulin was used as a loading control (right panel).

It is well known that β1 plays crucial roles in the regulation of cell migration and in intracellular cell adhesion signaling such as in the phosphorylation of FAK^19^. Using a standard wound healing assay, as expected, KO cells showed a significant decrease in cell migration, compared with the results seen in WT cells (Fig. 2A). Consistently, the phosphorylated levels of FAK and AKT were also decreased in KO cells, compared with the results seen in WT cells (Fig. 2B). As shown in Fig. 2C, WT cells showed high levels of α-SMA and marginal expression levels of E-cadherin, which were mesenchymal and epithelial makers, respectively. Interestingly, in agreement with the changes in morphology, KO cells showed greatly enhanced expression levels of E-cadherin and reduced levels of α-SMA. The phenotypes of KO cells, such as increased cell-cell adhesions, typical changes in mesenchymal and epithelial makers, and suppression of cell migration are reminiscent of a re-differentiation towards an epithelial phenotype, resembling mesenchymal-epithelial transition (MET), which is the reverse process of epithelial-mesenchymal transition (EMT). We next investigated whether transforming growth factor-β (TGF-β), a well-established physiological inducer of EMT, could overcome the requirements for β1 integrin. As shown in Fig. 2D, treatment with TGF-β normally induced the phosphorylation of Smad2, a specific signaling pathway of TGF-β, promoted the loss of E-cadherin and accelerated the induction of α-SMA. Although the TGF-β downstream pathways leading to EMT were retained, they did not result in morphological changes. These results suggested that β1 was crucial for the maintenance of mesenchymal phenotypes by stabilizing focal adhesions, which may also be required for cell survival.

### Effects of β1 on cell proliferation via a cell density-dependent manner

**Figure 3:**
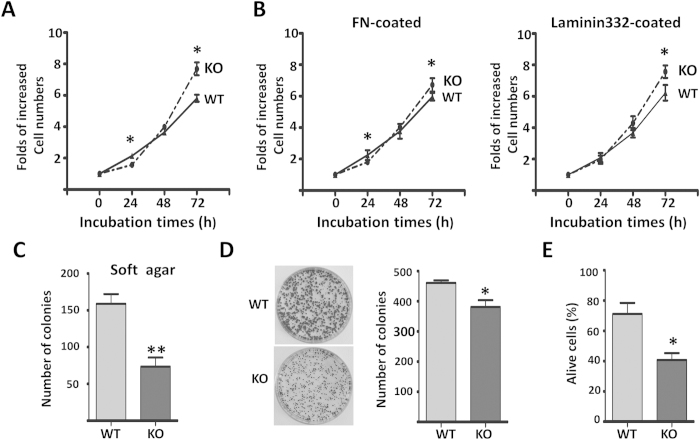
Effects of β1 on the proliferation and survival in MDA-MB-231 cells. (**A,B**) WT and KO cells were cultured on the none-coated (A) or FN, Laminin 332-coated (**B**) dishes, starved with serum-free DMEM for 24 h and then released with DMEM containing 10% FBS, the numbers of live cells were counted at the indicated times. Cell numbers were normalized to those at 0 h. Data are represented as the means ± s.e.m (n = 3) (*p < 0.05 by two-tail unpaired t-test). (**C**) WT and KO cells (500/per well) were grown for 16 days, then stained with crystal violet and the foci in each well were counted. Two representative wells are shown (*p < 0.05 by two-tail unpaired t-test). (**D**) WT and KO cells (500/per well) were cultured in the soft-agar plates for 18 days, the colonies were stained with crystal violet and counted, the quantitative data are presented as the means ± s.e.m from three independent experiments (**p < 0.01 by two-tail unpaired t-test). (**E**) WT and KO cells were cultured over a layer of 1% agarose in serum-free media for 72 h. Cells were then collected by centrifugation and stained with trypan blue. The percentage of live cells was calculated. The quantitative data are presented as the means ± s.e.m from three independent experiments (*p < 0.05 by two-tail unpaired t-test).

As described above, β1 had a strong impact on cell behaviors such as morphology and migration, so we further investigated the effect that the deletion of β1 had on these biological functions. Surprisingly, when compared with WT cells, the KO cells tended to reach a confluence under normal culture conditions (Fig. 3A). Considering the lower level of phosphorylated AKT in the KO cells, as shown in Fig. 2B, we hypothesized that changes in cell density might be critical for the regulation of cell proliferation. As shown in Fig. 3A, when cells were cultured at a lower density level in the first 24 h of incubation, the KO cells showed a significant decrease in cell proliferation, by comparison with the WT cells. However, once the KO cells reached a certain density, their cell proliferation ability was significantly increased, compared with the WT cells. We questioned whether the extracellular matrix could affect the anchorage-dependent cell growth, which would suggest that the dependence on cell densities could be the result of the concentration of ECM secreted from the cells, particularly under low-density conditions. Therefore, we checked the cell growth following a coating with ECMs. Interestingly, we found that an accumulation of laminin-332, rather than fibronectin, eliminated the difference between WT and KO cells under sparse conditions (Fig. 3B), which indicated that the up-regulation of β4 integrin can partially rescue the cell proliferation of KO cells. These data also suggested that cell survival signaling from β1 integrin might play an important role in cell proliferation when cells are under strict conditions. To confirm this notion, we then conducted a clonogenic assay to test the single-cell potential for survival and ‘unlimited’ cell division^20^. The KO cells consistently formed smaller and fewer foci compared with the WT cells (Fig. 3C). Furthermore, we compared the anchorage-independent cell growth of WT and KO cells via soft-agar assays. Anchorage-independent cell growth is thought to be closely related to cell survival signaling^21^. As expected, the KO cells formed fewer colonies than the WT cells (Fig. 3D). The abilities for cell survival were further examined under serum-free conditions after being cultured for 3 days, and we found that the deletion of β1 dramatically suppressed cell survival (Fig. 3E). Since the soft-agar and clonogenic assays were performed under low cell density culture conditions, these results strongly supported the results in Fig. 3A, which show that β1 affected cell proliferation in a cell density-dependent manner.

### Deletion of β1 suppressed tumorigenesis *in vivo*

**Figure 4:**
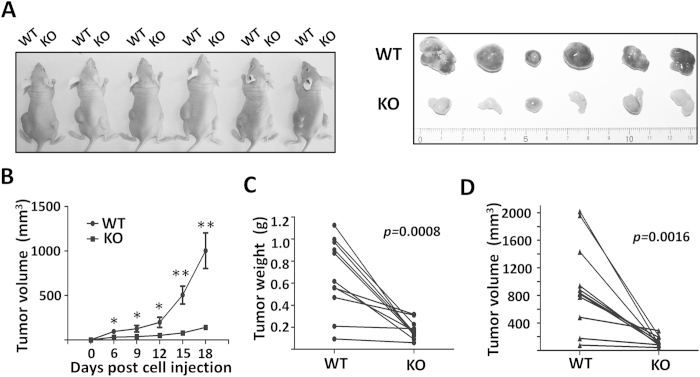
β1 suppressed xenograft tumor growth *in vivo*. (**A**) WT cells (1×10^6^) were injected subcutaneously into the left side of the flank and KO cells (1×10^6^) were injected into the opposite side of the same mice. Tumor volume was measured every 3 days after injection for 6 days until the tumor had grown to the approved size (**B**) (n = 11, *p < 0.05, **p < 0.01 by two-tail unpaired t-test). Tumors were dissected, weights (**C**) and volumes (**D**) were measured. Values are presented as the means ± s.e.m (n = 11, p values by two tail-paired t-test).

To further evaluate the impact of β1 on tumor growth, we examined tumorigenesis *in vivo* using a well-established xenograft tumor model. After subcutaneous injection of the WT and KO cells (1 × 10^6^) into the right and left flanks of nude mice, respectively, to allow tumor formation, we monitored tumor growth for 18 days. Compared with the WT group, the xenograft tumor formation in the KO group was significantly suppressed in terms of both weight and volume (Fig. 4A–D), which indicated that β1 played important roles in tumorigenesis *in vivo*.

### Activation of EGFR and cellular signaling were altered in the KO cells via a cell density-dependent manner

**Figure 5:**
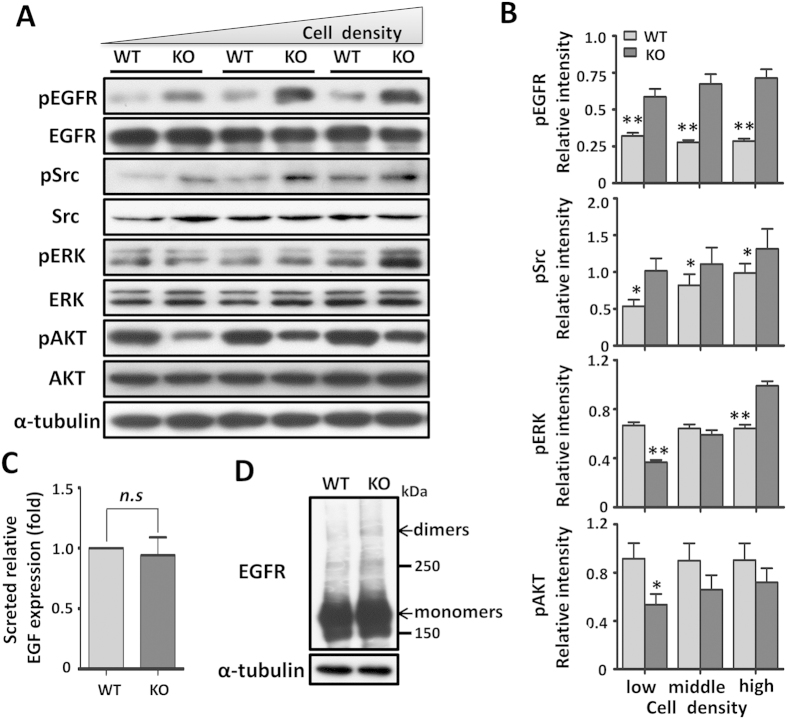
β1 regulated cell signaling with a cell density-dependent manner and was dispensable for EGFR dimer formation. (**A**) Low, middle and high densities of cell lysates were immunoblotted by anti-pEGFR, anti-EGFR, anti-pSrc, anti-Src, anti-pERK, anti-ERK, anti-pAKT and anti-AKT antibodies. α-Tubulin was used as a loading control. (**B**) Graphical representation of relative level of pEGFR, pSrc, pERK and pAKT in low, middle and high densities of MDA-MB-231 cells, respectively. Data are represented as the means ± s.e.m of three independent experiments (*p < 0.05, **p < 0.01 by two-tail unpaired t-test). (**C**) EGF concentration in the secreted media was analyzed by EGF ELISA. EGF levels of KO cells were normalized to WT cells. Data are represented as the means ± s.e.m (n = 3, n.s: no significance). (**D**) Cells were subsequently cross-linked using 5 mM Sulfo-EGS as chemical cross-linker on ice for 2 h and stopped with 10 mM Tris for 15 min. Cell lysates from those cells were subjected to SDS-PAGE, and then blotted with anti-EGFR antibody to detect EGFR monomers and dimers. α-Tubulin was used as a loading control.

As described above, the effects of β1 on cell proliferation were dependent on cell densities, we then examined the signaling events triggered by the cell-ECM interactions of WT and KO cells under different cell density conditions. Most of the studies regarding EGFR have focused on lung cancer, and the role of EGFR in breast carcinogenesis is still poorly established^22^,^23^. In recent studies, however, EGFR has assumed a relevant place in breast cancer etiology after its association with aggressive clinical behavior showing a characteristic pattern of metastatic dissemination to the lungs and brain^24^ and poor responses to conventional chemotherapies^25^. Src-family kinases are controlled by many cell surface receptors, including integrin receptors and protein-tyrosine kinases, which participate in pathways regulating cell proliferation and survival^26^. It was particularly interesting that under normal culture conditions the levels of phosphorylated EGFR and Src were significantly up-regulated in KO cells by comparison with WT cells (Fig. 5A), which suggested that expression of β1 suppressed EGFR activation and Src was involved in EGFR signaling in this cell line. By contrast, the activation of ERK appeared to happen in a cell density-dependent manner where decreased levels of phosphorylated ERK occurred at lower cell densities, and increased levels of phosphorylated ERK were found in higher cell densities in the KO cells, compared with those in WT cells. However, the levels of phosphorylated AKT were decreased in all cell culture densities in the KO cells, compared with the WT cells, which further strongly suggested that the expression of β1 played an important role in cell survival signaling. As the level of phosphorylated EGFR was increased in KO cells, we next investigated whether the increased levels of phosphorylated EGFR were due to excessive EGF secretion. As shown in Fig. 5C, media-secreted EGF was not affected by β1, thus confirming that activation of the EGFR in KO cells is a ligand-independent event. Dimerization is certainly a critical part of the mechanism during EGFR activation and EGF-induced signal transduction^27^. To study the mechanism for the deletion of β1-mediated EGFR activation, we compared EGFR dimerization in both types of cells. Cells were chemically cross-linked and then extracts were subjected to western blot testing to detect both EGFR monomers and dimers. During this testing, fewer EGFR dimmers were visualized in WT cells (Fig. 5D), suggesting that β1-KO cells facilitated EGFR activation.

### Restoration of the β1 gene rescued cell morphology, cell behaviors and EGFR activation in the KO cells

**Figure 6:**
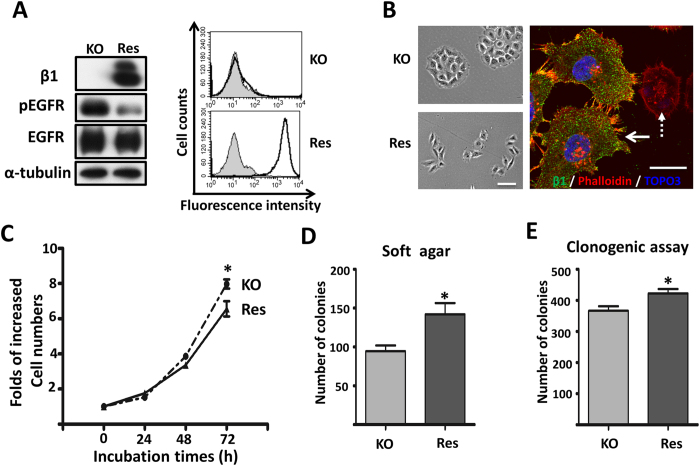
Forced expression of β1 rescued cell morphology and proliferation ability. (**A**) KO and KO-overexpressing β1 (Res) cells were immunoblotted by anti-β1, anti-pEGFR and anti-EGFR antibodies. α-Tubulin was used as a loading control (left panel). KO and Res cells were collected and incubated with (bold line) or without (grey shadow) anti-β1, followed by incubation with Alexa Fluor 647 goat anti-mouse IgG subjected to flow cytometry analysis (right panel). (**B**) Bright field pictures were taken to show representative cell morphology of KO and Res cells (left panel). Scale bar represents 50 μm. Res cells were stained with anti-β1, followed by incubation with fluorescent secondary antibody. Localization of F-actin was examined by staining with Alexa Fluor 546 phalloidin. The solid and dotted arrows indicated Res and KO cells, respectively. The bar denotes 20 μm (right panel). (**C**) KO and Res cells were starved with serum-free DMEM for 24 h and then released with DMEM containing 10% FBS, and the number of cells were counted at indicated time points. Cell numbers were normalized to those at 0 h. Values are the means ± s.e.m (n = 3, *p < 0.05 by two-tail unpaired t-test). (**D**) KO and Res cells (500/per well) were cultured in the soft-agar plates. After 18 days, the colonies were stained with crystal violet and the numbers of colonies in each plate were counted. The quantitative data are presented as the means ± s.e.m from three independent experiments (*p < 0.05 by two-tail unpaired t-test). (**E**) KO and Res cells (500/per well) were grown for 16 days and the foci in each well were counted. The quantitative data are presented as the means ± s.e.m from three independent experiments (*p < 0.05 by two-tail unpaired t-test).

Given the observation that the KO cells exhibited both aberrant cell morphology and cell growth ability, we restored β1 expression in the KO cells (Res) to examine whether it would rescue these phenotypes. The efficiencies of Res cells were confirmed by western blot and flow cytometry analysis (Fig. 6A). As expected, the EGFR activation was greatly suppressed in the Res cells, similar to the WT cells, by comparison with the KO cells (Fig. 6A). The aggregated cell morphology of the KO cells was reversed to a mesenchymal morphology, including filopodia formation and loss of cortical actin formation in the Res cells (Fig. 6B). In addition, cell proliferation was suppressed, while the ability of colony formation, both in soft agar and in culture dishes, was increased in the Res cells, compared with those in the KO cells (Fig. 6C–E). These results supported the notion that β1 expression dynamically regulates cell signaling to control cell proliferation and survival.

### EGFR tyrosine kinase inhibitor, AG1478, efficiently suppressed cell proliferation in KO cells or WT cells in the presence of anti-β1 antibody

**Figure 7:**
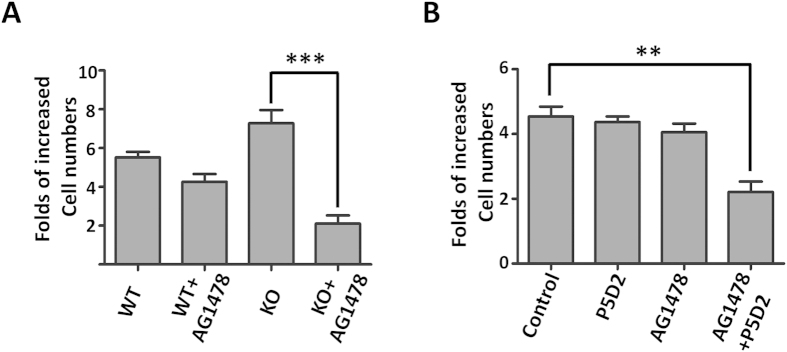
Treatment with AG1478 efficiently suppressed cell proliferation in KO cells. (**A**) After attachment for 24 h, WT and KO cells treated with 3 μM of AG1478 for 48 h, the number of live cells were counted. Cell numbers were normalized to those at 0 h. Data are represented as the means ± s.e.m (***p < 0.001 by two-tail unpaired t-test). (**B**) MDA-MB-231 cells were untreated, or treated with P5D2, AG1478, or P5D2 plus AG1478 for 48 h. Cell proliferation was evaluated by the number of live cells. Cell numbers were normalized to those at 0 h as 1. Data are represented as the means ± s.e.m (**p < 0.01 by two-tail unpaired t-test).

Different anti-EGFR agents, which include the EGFR tyrosine kinase inhibitor gefitinib, have shown an inhibition of the growth of human breast carcinoma cells^28^,^29^. However, clinical studies of gefitinib in breast cancer have resulted in few clinical responses and in a disease control rate of approximately 10%^30^. This observation indicates that resistance to gefitinib is a common phenomenon in breast cancer. AG1478 shares the same structural quinazoline backbone with gefitinib, and it has also shown an ability to inhibit the function of EGFR^31^. As described above, the differential activation of EGFR and its downstream pathways including Src, ERK and AKT were observed in the WT and KO cells. In the present study, we examined the influence of AG1478 on cell proliferation, which was inhibited in both types of cells, but the effect clearly was more effective in the KO cells than in the WT cells (Fig. 7A). Consistently, MDA-MB-231 cells treated with AG1478 combined with the inhibitory anti-β1 antibody (P5D2) and exhibited a significant decrease in cell proliferation, compared with a single treatment with either P5D2 or AG1478 alone (Fig. 7B). These results show that a combination treatment with anti-EGFR and anti-β1 agents exerts a synergistic inhibitory effect on cell growth, which may also solve the resistance problem for a single inhibitor in breast cancer treatment.

## Discussion

There is an increasing body of evidence implicating members of the integrin family as important signaling components involved in mammary tumorigenesis and progression^15^, since integrins play important roles in cell migration, cell proliferation and cell survival. In the present study, we performed a combined biochemical technique via the knockout and restoration of the β1 gene in MDA-MB-231 cells, a highly invasive breast cancer cell line, and found that β1 is not only important for cell migration and survival, it also regulates cell proliferation in a novel cell density-dependent manner. When cells are cultured under a relatively low degree of density, they exhibit β1-dependent growth. However, once cells reach a certain density, the effects of β1 are reversed, and then they suppress cell proliferation and EGFR signaling. Although the underlying mechanism remains unclear, at least two possible explanations could be concluded from the present study. One is the up-regulation of β4 integrin in KO cells, since previous researchers have already found that α6β4 integrin in MDA-MB-231 cells induces EGFR clustering^32^. The other possibility is that culturing under low density exposes cells to strict conditions, in which cell survival signaling from the β1-mediated cell-ECM interaction is essential for cell growth. When cells then reach a certain density, the cell survival signaling, which can be obtained from both cell-cell and cell-ECM interactions, is not important for cell proliferation. The significance of β1 in cell proliferation and survival remains controversial. Some studies have reported that the blocking of β1 inhibited cell proliferation and induced cell apoptosis^8^,^15^. However, other studies have shown that a down regulation of β1 expression promotes cell growth^10^,^33^. These discrepancies might be partly explained by the observations described above and other possibilities cannot be excluded, such as different approaches for blocking β1 and different cell lines, which express differential associations of β1 with divergent α subunits.

Our data clearly demonstrated that the expression of β1 suppressed cell proliferation and down-regulated the phosphorylation of EGFR, Src and ERK, but that the phosphorylation of Akt was up-regulated (Fig. 5). These results suggested that EGFR-mediated signaling, rather than integrin β1-mediated FAK phosporylation, plays a dominant effect on Src and ERK signaling, and that it regulates cell proliferation in KO cells. Because both integrin and EGFR can activate the PI3K/AKT signaling pathway, we can speculate that integrin β1-mediated activation could be a dominant PI3K/AKT signaling pathway in the cell. In fact, the inactivation of Akt in the KO cells was demonstrated by decreases in cell survival signaling and cell migration. In line with our observations, Mebratu, *et al.* reported that the blockage of β1 stimulated the MEK1/2-ERK1/2 signal pathway to promote cell proliferation^34^. These results suggested that an increase in cell proliferation ability could be a consequence of β1 inhibition. The restoration of the β1 gene in the β1-KO cells consistently and significantly suppressed EGFR activation (Fig. 6), which suggested the existence of a negative feedback loop between EGFR and β1. In fact, treatment with trastuzumab, a targeted therapy for HER2, is known to have resulted in an activation of β1-related signal pathways such as PI3K/AKT and ERK by circumventing the anti-cell proliferative activity of trastuzumab^35^,^36^. Taken together, these results indicate that the signaling from integrin and EGFR contributes to therapy resistance, a phenomenon that has also been observed in other cancer cells^37^. It is intriguing that the inhibitory effects of EGFR tyrosine kinase inhibitor AG1478 on cell proliferation were more effective in β1-KO cells than in WT cells, which suggested that a treatment combining anti-EGFR therapy with anti-integrin drugs wound be important for cancer therapy. Considering that the blockage of β1 may inhibit cell survival while simultaneously promoting cell proliferation, a combination treatment may be suitable for those post-treated with anti-EGFR drugs where cell survival signals are more essential than cell-growth signals for the treatment of cancer cells. The inhibitory effects of integrins on cell growth have also been confirmed by other studies; a deletion of α1β1 integrin enhanced EGFR cellular signaling through a clustering of EGFR^18^; additionally, the targeting of α2β1 promoted cell growth in some breast cancer cells^38^. Here, however, we could not exclude the possible involvement of β4 integrin, since its expression level was significantly increased in KO cells (Fig. 1). By cooperation with RTKs, α6β4 is known to positively regulate cell proliferation. Specifically, α6β4 can induce both EGFR and ErbB-2 clustering, which then promotes the proliferation of MDA-MB-231 cells^32^,^39^. Further investigation is obviously needed to elucidate the mechanistic roles of the reciprocal regulation between integrin and EGFR.

It is well known that EMT is an important malignant phenotype characterized by a loss of the cell–cell junction and the acquisition of cell migratory and invasive behavior in breast cancer^6^,^40^. In the present study, KO cells exhibited disruption of focal adhesions, stabilized cell-cell adhesion and typical changes in EMT markers to show a malignant phenotype reversion, even after TGF-β stimulation, which indicated that the malignant phenotype exhibited by MDA-MB-231 cells was primarily mediated by β1 integrin. β1 integrin is also necessary for induction into the murine 4 T1 cell, which is another invasive mammary cell line^6^. This is reasonable since β1 plays a crucial role in the changing of cell shape and in the forming of invasive protrusions during the cell migration process^41^,^42^,^43^. However, the GE11 cell, a β1-deficient normal cell line, is responsible for TGF-β stimulation, which can induce EMT-like changes^43^,^44^. These discrepancies could be explained by the fact that the requirement for β1 during malignancy progression is dependent on cell types, particularly in mammary cells. In fact, the balanced interactions between cell-ECM, cell-cell, and their dynamic regulations are vital for physiologic and pathologic events, and altered interactions with ECM have been observed in mammary tumor development^17^.

In conclusion, the current study clearly showed that integrin β1 dynamically regulates cell proliferation and cellular signaling. Basically, the expression of β1 negatively regulates EGFR activation. Considering the importance of β1 in cell survival and cell migration, a combination treatment with anti-EGFR and anti-β1 drugs is recommended as a very useful therapy for breast cancer.

## Methods

### Antibodies and reagents

The experiments were performed using the following antibodies: Antibody against human integrin β1 subunit (P5D2) was from Developmental Studies Hybridoma Bank, University of Iowa; Mouse mAb against Smad2, rabbit mAbs against EGFR, p-EGFR, ERK1/2, p-ERK1/2, AKT, p-AKT, p-Src, and p-Smad2 were from Cell Signaling Technology; rabbit pAb to β4 integrin and goat antibody against α3 integrin were from Santa Cruz Biotechnology (Santa Cruz, CA); mouse mAbs against β1 integrin, α5 integrin, β4 integrin, αv integrin, FAK, p-FAK, and rat antibody against α6 integrin were from BD Biosciences; mAb against α-tubulin and α-SMA were from Sigma; mouse mAbs against Src was from upstate biotechnology. Alexa Fluor^®^ 488 and 647 goat anti-mouse IgG was obtained from Invitrogen (Life Technologies). The peroxidase-conjugate goat antibody against mouse, rabbit and goat IgG were obtained from Chemicon and Cell Signaling Technology. The TO-PRO-3 was from Molecular Probes; the selective EGFR blocker Tyrphostin AG1478, fibronectin and laminin-332 were obtained from Sigma; the Sulfo-EGS was from Thermo Scientific; and, the Quantikine Human EGF Immunoassay kit was from R&D Systems.

### Cell culture

The 293T and MDA-MB-231 cell lines were purchased from RIKEN and ATCC, respectively. Cells were maintained at 37 °C in Dulbecco’s modified Eagle’s medium (DMEM), supplemented with 10% fetal bovine serum (FBS), under a humidified atmosphere containing 5% CO_2_, except for the virus production. β1-KO and rescue cells, as generated by the process described bellow, were also maintained in DMEM.

### Generation of CRISPR/Cas9-based β1-KO MDA-MB-231 cells

The CRISPR/Cas9-based β1-KO MDA-MB-231 cells were established as described previously^45^. Briefly, the sgRNA-specifying oligo sequences spanning human β1 integrin exon 2 (CACCGGAGGAATGTTACACGGCTGC-forward; AAACGCAGCCGTGTAACATTCCTCC-reverse), which were chosen from the human KO library sgRNAs^46^ were cloned into the pSpCas9 (BB)-2A-GFP (Addgene plasmid ID: 48138) vector. The plasmid was electroincorporated into the MDA-MB-231 cells according to the manufacturer’s instructions (Amaxa^®^ cell line Nucleofector^®^ kitV). After 72 h of transfection, GFP-positive cells were sorted using the FACSAria II (BD Bioscience). Following about 10 days culture, the β1-negative and GFP-negative cells were sorted another five times. The β1-KO cells were defined by flow cytometry and western blot analyses as described below.

### Establishment of β1 rescued MDA-MB-231 cells

The vector of pENTR-D-Topo-β1 was previously established in our laboratory^47^. We then used a Gateway^TM^ cloning System kit (Invitrogen) for getting the expression vectors. Briefly, a LR clonase reaction (Invitrogen) was used to transfer the cDNAs of β1 from the entry vectors into CSII-EF-Rfa. The CSII-EF-β1 was cotransfected with pCAG-HIVgp and pCMV-VSV-G-RSV-Rev into 293T cells. After infection for 48 h, the virus media was collected. The KO cells were infected with the resultant virus for 72 h, and the β1 positive cells were selected using the FACSAria II (BD Biosciences) twice. The stable cell line was used in subsequent studies.

### Western blot (WB)

Cells of high, middle and low densities were seeded in 60-mm dishes overnight, then washed with ice-cold PBS and lysed in lysis buffer (20 mM Tris-HCl pH 7.4, 150 mM NaCl, 1% Triton X-100) with protease inhibitors and phosphatase inhibitors (Nacalai Tesque, Kyoto, Japan) for 30 min. After centrifugation, the supernatants were collected and protein concentrations were determined using a BCA protein assay kit (Pierce). The protein lysates were subjected to SDS-PAGE. After electrophoresis, the proteins were transferred to a PVDF membrane (Millipore). The membrane was detected with primary and secondary antibodies, and the proteins were visualized by Immobilon Western Chemiluminescent HRP Substrate (Millipore) according to the manufacturer’s instructions. Uncropped scans for the main Western blots were shown in Supplementary information.

### Flow cytometric analysis

Cells were grown to about 90% confluency and then detached from the culture dishes using trypsin containing 1 mM EDTA, washed with ice cold PBS and stained with the primary antibodies, followed by incubation with Alexa Fluor 647 goat anti-mouse IgG (Invitrogen) for 60 min on ice, respectively. Finally, the cells were washed three times with PBS and analyzed via FACSCalibur flow cytometry (BD Biosciences).

### Immunofluorescence staining

Cells were cultured on a glass-bottom dish, washed with PBS and fixed with ice-cold methanol and permeabilized with 0.2% Triton-X-100. Antibodies against β1 (P5D2), pFAK and β4 were used followed by incubation with anti-mouse Alexa Fluor 488 secondary antibodies (Invitrogen) and Alexa Fluor 546 phalloidin (Invitrogen). Fluorescence images were observed via confocal microscopy using a FluoView FV1000 (Olympus, Tokyo, Japan).

### *In vitro* wound-healing assay

Cells were seeded in a 6-well plate and grown to a confluent monolayer. A “scratch” with a p200 pipet tip was made through the cell layer. After washing with PBS, DMEM containing 2% FBS was added in each well. Wounded areas were photographed under a light microscope at 10× objective after 24 h. All experiments were repeated three times.

### RT-PCR for mRNA expression analysis

Total RNA was treated with TRIzol reagent (Invitrogen), and 1 μg of total RNA was reverse-transcribed using a SuperScript III first-strand synthesis system (Invitrogen) according to the manufacturer’s instructions. The sequences of the primers used for the PCR (sense and antisense, respectively) were as follows: E-cadherin, 5′-ACGCATTGCCACATACA-3′ and 3′-CGTTAGCCTCGTTCTCA-5′; α-SMA, 5′-CCAGCGACCCTAAAGCTTCC-3′ and 3′-ACCATCACCCCCTGATGTCTG-5′. Glyceraldehyde-3-phosphate dehydrogenase (GAPDH) mRNA was used as a control.

### Cell growth assay

Cells (3 × 10^4^) were seeded into 60-mm dishes overnight and then serum-starved for 24 h. After starvation, the cells were released with complete media with or without tyrphostin AG1478 (3 μM), an inhibitor of EGFR tyrosine kinase activity, or P5D2 (3 μg), an inhibitory anti-β1 antibody for 48 h. Cells in the same area were photographed in phase contrast at the indicated times, then the numbers of living cells were counted. Cell numbers were normalized to those at 0 h and statistically analyzed.

### Soft agar assay

Cells were mixed in 0.33% agarose and layered on top of prepared 0.5% base agar plates in DMEM containing 10% FBS. The agar plates were incubated at 37 °C and the cultured media were changed twice weekly. After culture for 20 days, the plates were stained with 0.005% crystal violet, and then colonies were counted from three independent dishes of each sample.

### Clonogenic assay

Cells (500/per well) were seeded in a 6-well plate in complete media, incubated at 37 °C and the cultured media were changed twice weekly. After culture for 18 days, the foci were stained with 0.005% crystal violet, and colonies containing more than 50 cells were counted from three independent wells of each sample.

### Cell survival in suspension

A 1% base agar was prepared in complete media on the bottom of a 60 mm dish. Cells were collected, washed and plated on top of the agar dishes, and then cultured for 3 days in serum-free media. Cells were collected by centrifugation, stained with trypan blue, and the living cells were counted.

### Xenograft assay

The bilateral flanks of the five-week-old female nude mice (Charles River Laboratories, Japan) were injected subcutaneously with indicated cells (1 × 10^6^). Tumor growth was monitored every 3 days. The tumor tissues were harvested after 18 days and their volumes and weights were measured. All experiments involving animals were performed according to protocols approved by the Tohoku Pharmaceutical University Research Ethics Board.

### ELISA

WT and KO cells were seeded on uncoated 6-well plates and after 24 h the cells were incubated with serum-free media. After 72 h, the media was collected, and stored at −80 °C until assay. EGF in the media was assayed using a Quantikine^®^ Human EGF Immunoassay kit (R&D Systems), according to a procedure described by the manufacturer.

### Chemical cross-linking of EGFR in intact cells

Cells were incubated with 5 mM Sulfo-EGS dissolved in PBS on ice for 2 h and then stopped using 10 mM Tris for 15 min. Cells were then solubilized with lysis buffer and subjected to anti-EGFR to detect both EGFR monomers and dimers.

### Statistical analysis

Statistical analyses were performed via a Student’s *t* test, using GraphPad Prism5. Results are presented as the mean ± s.e.m. Statistical significance was defined as *p* < 0.05 (*p < 0.05; **p < 0.01; ***p < 0.001).

## Additional Information

**How to cite this article**: Hou, S. *et al.* Distinct effects of β1 integrin on cell proliferation and cellular signaling in MDA-MB-231 breast cancer cells. *Sci. Rep.* **6**, 18430; doi: 10.1038/srep18430 (2016).

## Supplementary Material

Supplementary Information
