# Supplementary material for: Distinct effects of β1 integrin on cell proliferation and cellular signaling in MDA-MB-231 breast cancer cells
Source: Sci Rep. 2016 Jan 5;6:18430. doi: 10.1038/srep18430 (PMC4700444; doi:10.1038/srep18430)
Supplement: Supplementary Information [file srep18430-s1.pdf]

Supplementary Information

Distinct effects of β1 integrin on cell proliferation and cellular signaling in MDA-MB-231 breast cancer cells

Sicong Hou[1], Tomoya Isaji[1], Qinglei Hang[1], Sanghun Im[1], Tomohiko Fukuda[1] & Jianguo Gu[1]

[1]Division of Regulatory Glycobiology, Institute of Molecular Biomembrane and Glycobiology, Tohoku Pharmaceutical University, Sendai, Miyagi, 981-8558, Japan.

Correspondence and requests for materials should be addressed to J.G. (e-mail: jgu@tohoku-pharm.ac.jp); Tel: +81-22-727-0216; Fax: +81-22-727-0078

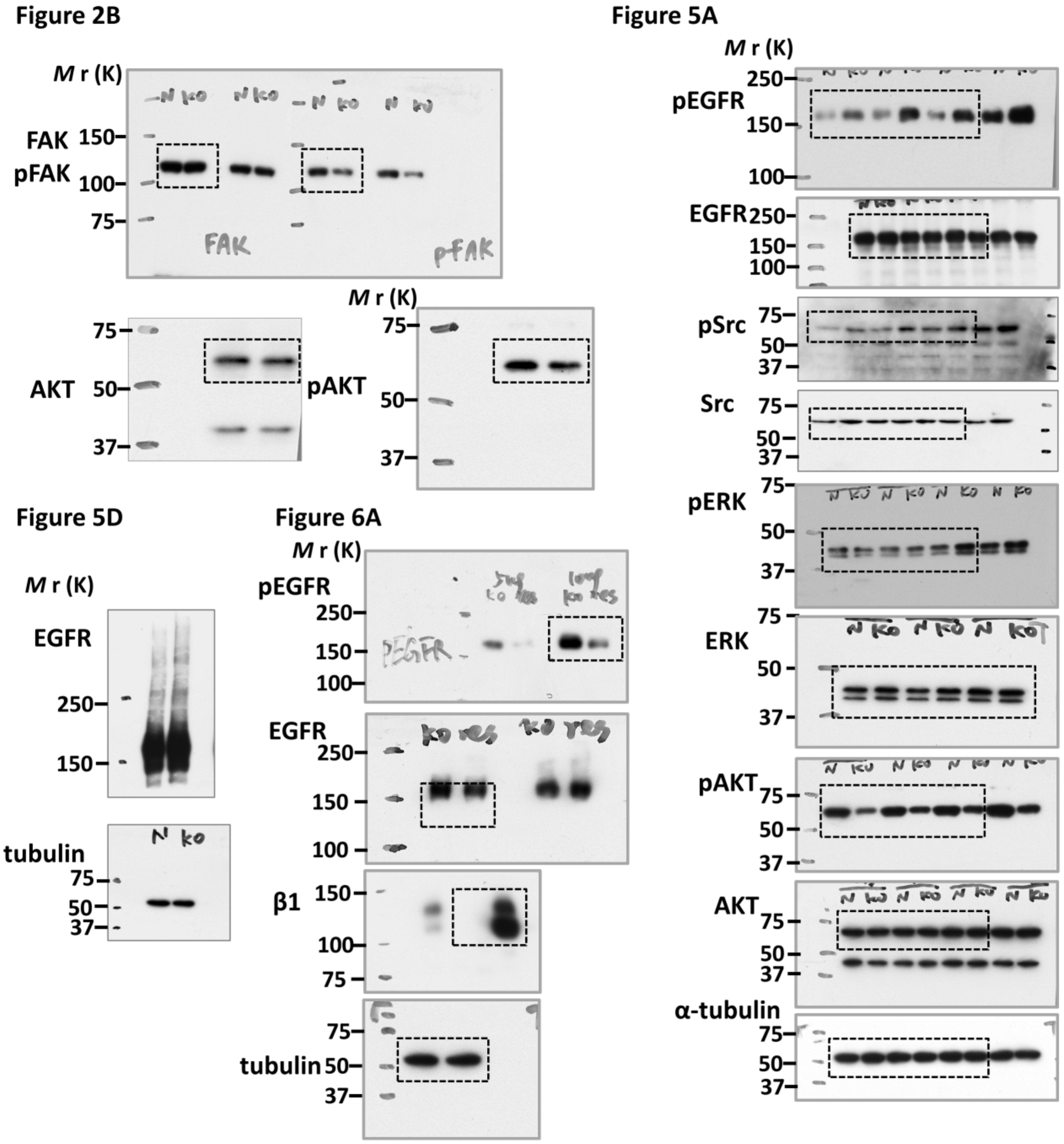

Supplementary Figure. Uncropped scans for the main western blots illustrated in Fig.2, 5 and 6.

Supplementary Figure: Uncropped, full-size scans of western blots

Uncropped scans for the main western blots illustrated in Fig.2, 5 and 6.
